# Synthesis of 12-aminododecenoic acid by coupling transaminase to oxylipin pathway enzymes

**DOI:** 10.1007/s00253-023-12422-6

**Published:** 2023-02-21

**Authors:** Anna Coenen, Manuel Ferrer, Karl-Erich Jaeger, Ulrich Schörken

**Affiliations:** 1 Faculty of Applied Natural Sciences, TH Köln University of Applied Sciences - Leverkusen Campus, Leverkusen, Germany; 2 ICP, CSIC, Madrid, Spain; 3 Institute of Molecular Enzyme Technology, Heinrich Heine University Düsseldorf, Forschungszentrum Jülich, Jülich, Germany; 4 Institute of Bio- and Geosciences IBG-1: Biotechnology, Forschungszentrum Jülich GmbH, Jülich, Germany

**Keywords:** Transaminase, 12-aminododecenoic acid, Polyamide, Nylon-12, Enzyme cascade

## Abstract

**Abstract:**

Biobased polymers derived from plant oils are sustainable alternatives to petro based polymers. In recent years, multienzyme cascades have been developed for the synthesis of biobased ω-aminocarboxylic acids, which serve as building blocks for polyamides. In this work, we have developed a novel enzyme cascade for the synthesis of 12-aminododeceneoic acid, a precursor for nylon-12, starting from linoleic acid. Seven bacterial ω-transaminases (ω-TAs) were cloned, expressed in *Escherichia coli* and successfully purified by affinity chromatography. Activity towards the oxylipin pathway intermediates hexanal and 12-oxododecenoic acid in their 9(*Z*) and 10(*E*) isoforms was demonstrated for all seven transaminases in a coupled photometric enzyme assay. The highest specific activities were obtained with ω-TA from *Aquitalea denitrificans* (TR_AD_), with 0.62 U mg^−1^ for 12-oxo-9(*Z*)-dodecenoic acid, 0.52 U mg^−1^ for 12-oxo-10(*E*)-dodecenoic acid and 1.17 U mg^−1^ for hexanal. A one-pot enzyme cascade was established with TR_AD_ and papaya hydroperoxide lyase (HPL_CP-N_), reaching conversions of 59% according to LC-ELSD quantification. Starting from linoleic acid, up to 12% conversion to 12-aminododecenoic acid was achieved with a 3-enzyme cascade comprising soybean lipoxygenase (LOX-1), HPL_CP-N_ and TR_AD_. Higher product concentrations were achieved by the consecutive addition of enzymes compared to simultaneous addition at the beginning.

**Key points:**

• *Seven ω-transaminases converted 12-oxododecenoic acid into its corresponding amine.*

• *A three-enzyme cascade with lipoxygenase, hydroperoxide lyase, and ω-transaminase was established for the first time.*

• *A one-pot transformation of linoleic acid to 12-aminododecenoic acid, a precursor of nylon-12 was achieved.*

**Graphical Abstract:**

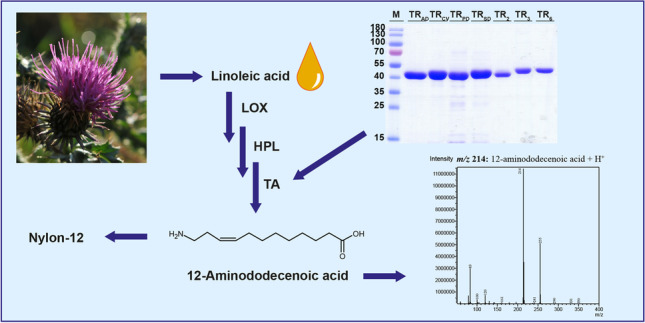

**Supplementary Information:**

The online version contains supplementary material available at 10.1007/s00253-023-12422-6.

## Introduction

Polyamides are important industrial polymers. Among them, nylon-12 exhibits properties that are centered between short-chain aliphatic nylons (e.g. nylon-6) and high-molecular-weight polymers (e.g., polyethylene). Nylon-12, as a high-performance polymer with good heat, UV and chemical resistance, is manufactured by ring opening polymerization of ω-laurolactam (Ladkau et al. 2016). Laurolactam is synthesized in a complex reaction cascade starting from the trimerization of petro-derived butadiene followed by cyclododecane oxime synthesis and Beckmann rearrangement (Karau et al. 2015). An increasing demand exists to replace fossil resources with renewable materials, forcing a switch in polymer production. For polyamide precursors, engineered whole-cell biocatalysts were used for their synthesis from fatty acids in multistep enzyme cascades. For example, 11-aminoundecanoic acid, a precursor for nylon-11, was obtained from 12-hydroxystearic acid using alcohol dehydrogenase, Baeyer–Villiger monooxygenase, esterase, and ω-transaminase (ω-TA) (Song et al. 2014). Furthermore, a whole-cell biocatalyst was designed using the alkane monooxygenase AlkBGT from *Pseudomonas putida* GPo1 and the ω-TA CV2025 from *Chromobacterium violaceum* for the synthesis of 12-aminododecanoic acid methyl ester from methyl laurate (Schrewe et al. 2013). The cascade was further improved by implementation of an alanine regeneration system to supply sufficient amounts of cosubstrate, overexpression of the outer membrane protein AlkL for substrate uptake and implementation of alcohol dehydrogenase AlkJ for increased alcohol oxidation (Ladkau et al. 2016; Ge et al. 2020).

**Fig. 1:**
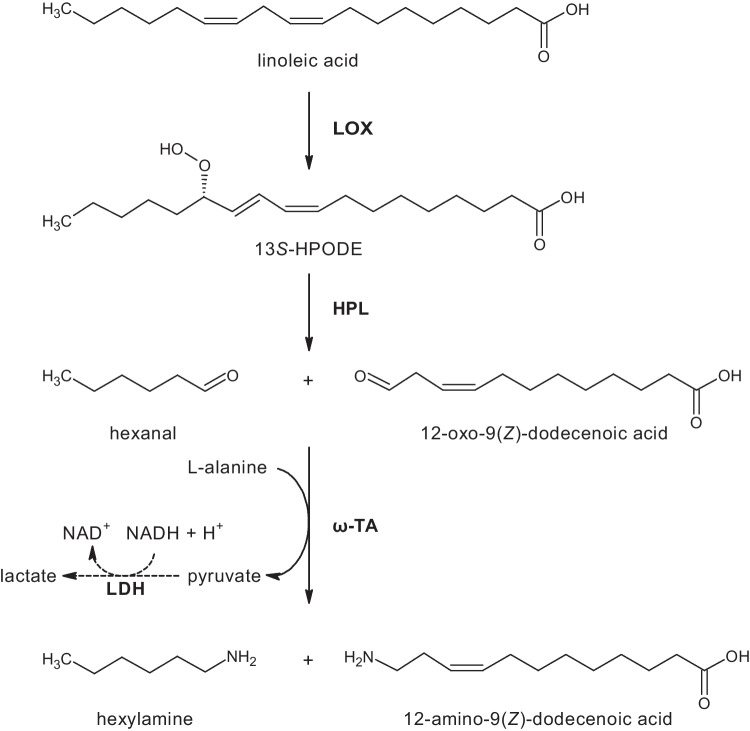
Reaction scheme showing the transformation of linoleic acid with oxylipin pathway enzymes lipoxygenase and hydroperoxide lyase coupled to ω-transaminase. For the synthesis of hexylamine and 12-amino-9(*Z*)-dodecenoic acid. Dotted lines indicate the coupled photometrical enzyme assay with lactate dehydrogenase and NADH. Abbreviations are lipoxygenase (LOX), hydroperoxide lyase (HPL), ω-transaminase. (ω-TA), lactate dehydrogenase (LDH), and 13*S*-hydroperoxy-9(*Z*),11(*E*)-octadecadienoic acid (13*S*-HPODE)

A disadvantage of the methyl laurate route towards nylon-12 is the utilization of tropical oils, which are the only source of natural lauric acid in large quantities. In contrast, linoleic acid is present in safflower or sunflower oil at high concentrations. In this work, we targeted a novel enzyme cascade for the synthesis of 12-aminododecenoic acid from linoleic acid (Fig. 1). For this purpose, lipoxygenase (LOX) and hydroperoxide lyase (HPL) originating from the oxylipin pathway were coupled to a transaminase reaction. In previous work from our group, 12-oxo-9(*Z*)-dodecenoic acid and hexanal were obtained from safflower oil in an enzyme cascade utilizing lipase, LOX, and HPL (Coenen et al. 2022). To date, the application of LOX and HPL has mainly focused on the green note synthesis of C6- and C9-aldehydes and their corresponding alcohols (Gigot et al. 2012; Vincenti et al. 2019; Stolterfoht et al. 2019). Otte et al. (2013) used 9-specific LOX and HPL for the synthesis of 9-oxononanoic acid. The integration of LOX and HPL into an *E. coli* host enabled the synthesis of the corresponding bifunctional azelaic acid employing endogenous oxidoreductases. The application of this intermediate as a polyester or polyamide building block was proposed (Otte et al. 2014).

ω-TA from *C*. *violaceum* has previously been shown to accept 12-oxododecanoic acid as a substrate (Schrewe et al. 2013), but the corresponding unsaturated 12-oxo-9(*Z*)-dodecenoic acid has not yet been tested as a ω-TA substrate. Therefore, we analyzed seven ω-TAs for their potential to synthesize 12-amino-9(*Z*)-dodecenoic acid. In addition to ω-TA from *C. violaceum* (TR_CV_) (Kaulmann et al. 2007), we chose enzymes from *Paracoccus denitrificans* (TR_PD_) (Rausch et al. 2013), and from uncultured bacteria most likely assigned to *Acidihalobacter* genus (TR_2_) and *Rhodobacteraceae* family (TR_3_ and TR_6_) (Coscolín et al. 2019). Additionally, new ω-TAs from *Aquitalea denitrificans* (TR_AD_) and *Sulfitobacter delicatus* (TR_SD_) were selected based on sequence homology analysis. The best-performing ω-TA was coupled in a one-pot reaction with lipoxygenase LOX-1 from *Glycine max* and hydroperoxide lyase HPL_CP-N_ from *Carica papaya* to demonstrate the feasibility of linoleic acid-based synthesis of nylon-12 precursors.

## Materials and methods

### Reagents

The 12-aminododecanoic acid standard was purchased from Alfa Aesar (Haverhill, MA, USA), while the 12-oxo-9(*Z*)-dodecenoic acid and 12-oxo-10(*E*)-dodecenoic acid standards were purchased from Larodan (Solna, Sweden). Pyridoxal-5-phosphate monohydrate (PLP) was obtained from Acros Organics, Thermo Fisher Scientific (Waltham, MA, USA). Hexanal, hexylamine, soybean LOX-1, and L-lactate dehydrogenase (LDH) were supplied from Sigma Aldrich (St. Louis, MO, USA). Linoleic acid was obtained from Thermo Fisher Scientific (Waltham, MA, USA). β-Nicotine amide adenine dinucleotide disodium salt (NADH), isopropyl β-d-1-thiogalactopyranoside (IPTG), l-alanine, imidazole, ampicillin sodium salt, and kanamycin sulfate were purchased from Carl Roth (Karlsruhe, Germany). 13S-Hydroperoxy-9(*Z*),11(*E*)-octadecadienoic acid (13*S*-HPODE) was prepared from linoleic acid by a peroxidation reaction with LOX-1 from *Glycine max* as described previously (Gala Marti et al. 2021). All other chemicals were from Sigma-Aldrich (St. Louis, MO, USA), Thermo Fisher Scientific (Waltham, MA, USA) or Carl Roth (Karlsruhe, Germany).

### Bioinformatic analysis

Putative novel transaminases homologous to ω-TA from *C. violaceum* CV2025 (TR_CV_: accession number WP_011135573.1) were identified with the Basic Local Alignment Search Tool (BLAST) (Altschul et al. 1990)*.* We selected two new transaminases from *A.* *denitrificans* (TR_AD_: WP_159877958.1) and *S. delicatus* (TR_SD;_ accession number WP_093738538.1) and compared them to the previously described transaminases from *P.* *denitrificans* (TR_PD_: accession number ABL72050.1), *Acidihalobacter* sp. (TR_2_: accession number MH588437), and uncultured *Rhodobacteraceae* bacteria (TR_3_: accession number MF158202 and TR_6_: accession number MF158205). A multiple sequence alignment of ω-TAs was performed with Clustal Omega (Sievers et al. 2011). A phylogenetic tree was constructed based on the neighbor-joining algorithm with a bootstrap value of 1000 and was generated with ClustalX (Thompson et al. 1997) and NJPlot (Perrière and Gouy 1996).

### Cloning and expression of enzymes

The sequences coding for the transaminases TR_AD_, TR_CV_, TR_PD_, and TR_SD_ were codon-optimized by BioCat GmbH (Heidelberg, Germany) for synthesis in *E. coli*. The optimized sequences *tr*_AD_ (accession number: OP866794), *tr*_CV_ (accession number: OP866795), *tr*_PD_ (accession number: OP866796), and *tr*_SD_ (accession number: OP866797) were cloned into the pET-21b( +) vector (Table 1). A sequence coding for a C-terminal hexahistidine tag (His6) was added for affinity purification. Chemically-competent *E. coli* BL21(DE3) cells were transformed with the respective vectors by heat shock at 42 °C for 90 s according to Hanahan (1983) and protein expression was carried out. Shaking flasks with baffles were inoculated with 2% (v/v) of an overnight culture, and cells were grown in terrific broth (TB) containing 100 µg ml^−1^ ampicillin at 37 °C until the OD_600_ reached 0.7–1. Then, cell expression was induced with 1 mM IPTG, and the temperature was lowered to 20 °C. Cells were cultured for 24 h before they were harvested by centrifugation at 4500 × *g* for 15 min at 4 °C. The supernatant was discarded, and the cell pellets were frozen at − 20 °C until further use.

**Table 1:** Bacterial strains and vectors used in the experiments

	Description	Reference
Bacterial strain		
*Escherichia coli* BL21(DE3)	*E. coli* str. B F^–^ *ompT gal dcm lon hsdS*_*B*_(*r*_*B*_^–^*m*_*B*_^–^) λ(DE3 [*lacI lacUV5*-*T7p07 ind1 sam7 nin5*]) [*malB*^+^]_K-12_(λ^S^)	Studier and Moffatt 1986
*E. coli* MC1061	*Δ(araA-leu)7697*, *[araD139]*_*B/r*_, *Δ(codB-lacI)3*, *galK16*, *galE15*(GalS), *λ*^*−*^, *e14-*, *mcrA0*, *relA1*, *rpsL150*(strR), *spoT1*, *mcrB1*, *hsdR2*	Casadaban and Cohen 1980
Vectors		
pET-21b( +)	Expression vector, Amp^R^	Merck (Darmstadt, Germany)
pET-21b::His*tr*_AD_	Expression vector for *tr*_AD_ from *Aquitalea denitrificans* with sequence for His6 tag, Amp^R^	This study
pET-21b::His*tr*_CV_	Expression vector for *tr*_CV_ from *Chromobacterium violaceum* with sequence for His6 tag, Amp^R^	This study
pET-21b::His*tr*_PD_	Expression vector for *tr*_PD_ from *Paracoccus denitrificans* with sequence for His6 tag, Amp^R^	This study
pET-21b::His*tr*_SD_	Expression vector for *tr*_SD_ from *Sulfitobacter delicatus* with sequence for His6 tag, Amp^R^	This study
pRhokHi-2::His*tr*_2_	Expression vector for *tr*_2_ from *Acidihalobacter* sp. with sequence for His6 tag; Kan^R^	Coscolín et al. 2019
pBXCH::His*tr*_3_	Expression vector for *tr*_3_ from *Rhodobacteraceae* bacterium with sequence for His6 tag; Amp^R^	Coscolín et al. 2019
pBXCH::His*tr*_6_	Expression vector for *tr*_6_ from *Rhodobacteraceae* bacterium with sequence for His6 tag; Amp^R^	Coscolín et al. 2019
pET-28a::His*hpl*_CP-N_	Expression vector for *hpl*_CP-N_ from *Carica papaya* with sequence for His6 tag, Kan^R^	Coenen et al. 2022

*Kan*^*R*^ kanamycin resistance, *Amp*^*R*^ ampicillin resistance

The transaminases TR_2_, TR_3_, and TR_6_, which were cloned into pRhokHi-2 (for TR_2_) and pBXCH (for TR_3_ and TR_6_), were expressed in *E. coli* MC1061 as described previously (Coscolín et al. 2019). Hydroperoxide lyase from *C. papaya* (HPL_CP-N_) was expressed in *E. coli* BL21(DE3) using the pET-28a( +) expression vector as described previously (Coenen et al. 2022).

### Protein purification

All transaminases were C-terminally His6-tagged and purified by metal affinity chromatography. For purification of TR_AD_, TR_CV_, TR_PD_, and TR_SD_, cell pellets from a 50-ml culture were suspended in 10 ml 50 mM potassium phosphate buffer pH 7.5 containing 500 mM NaCl and 40 mM imidazole. The suspensions were incubated on ice for 1 h before the cells were sonicated seven times for 15 s to obtain the crude extract (CE). The soluble fraction (SF) was separated after centrifugation of the CE for 45 min at 21,000 × *g* at 4 °C and loaded onto a HisTrap™ FF column (Cytiva, Marlborough, MA, USA). Nonspecifically bound proteins were removed by washing with 50 mM potassium phosphate buffer pH 7.5 with 500 mM NaCl and 40 mM imidazole, and the enzyme was eluted with 250 mM imidazole. The buffer was exchanged to 50 mM potassium phosphate pH 7.5 with 50 mM NaCl using a HiTrap® Desalting column (Cytiva, Marlborough, MA, USA). The eluted protein fractions were concentrated with Pierce™ Protein Concentrators PES, 10 K MWCO from Thermo Fisher Scientific (Waltham, MA, USA). The His6-tagged transaminases TR_2_, TR_3_, and TR_6_ were purified by affinity chromatography as described by Coscolín et al. (2019), and HPL_CP-N_ was purified as described by Coenen et al. (2022). Protein concentrations were measured with Bradford reagent (Bradford 1976), and the purification process was monitored with sodium dodecyl sulfate–polyacrylamide gel electrophoresis (SDS-PAGE) (Laemmli 1970).

### Preparation of 12-oxododecenoic acid

12-Oxo-9(*Z*)-dodecenoic acid was obtained from 13*S*-HPODE by a hydroperoxide lyase reaction using HPL_CP-N_. Reaction mixtures were prepared with 5 mM 13*S*-HPODE and 20 U ml^−1^ HPL_CP-N_ in 50 mM potassium phosphate buffer pH 6 with 1 M NaCl for 15 min at 22 °C. The reaction products (12-oxo-9(*Z*)-dodecenoic acid and hexanal) were extracted by solvent extraction with methyl tert-butyl ether (MTBE). Due to the different volatilities of the substances, the solvent and hexanal could be evaporated with a vacuum concentrator, while 12-oxo-9(*Z*)-dodecenoic acid remained in the reaction vial. 12-Oxo-9(*Z*)-dodecenoic acid was dissolved in ethanol and frozen at − 80 °C until further use. The amount and purity of 12-oxo-9(*Z*)-dodecenoic acid were confirmed by GC analysis as described previously (Coenen et al. 2022). The remaining hexanal concentration was less than 1.5% compared to 12-oxo-9(*Z*)-dodecenoic acid.

### Transaminase activity assay

Transaminase activity was measured in triplicate in a coupled photometric enzyme assay with lactate dehydrogenase (LDH) and NADH in a volume of 1 ml. Reactions were carried out in a cuvette containing 10 µl ω-TA solution, 10 µl of a 50 U ml^−1^ LDH solution (Sigma Aldrich, St. Louis, MO, USA), 0.1 mM substrate, 10 mM l-alanine and 0.1 mM pyridoxal-5-phosphate (PLP) in 50 mM potassium phosphate buffer pH 7.5 with 50 mM NaCl. 12-Oxo-9(*Z*)-dodecenoic acid, 12-oxo-10(*E*)-dodecenoic acid, or hexanal were used as substrates. Reactions were started by the addition of NADH to a concentration of 0.1 mM, and the decrease in absorbance was measured at 340 nm for 300 s at 22 °C. The extinction coefficient of NADH of 6220 M^−1^ cm^−1^ was used to determine the volumetric activity, and specific activities were calculated with Microsoft Excel based on the protein concentrations of the purified ω-TAs. Reactions without substrate addition were set up as negative control to confirm the assay. One unit is defined as the amount of enzyme that catalyzes the amination of 1 µmol of substrate (12-oxo-9(*Z*)-dodecenoic acid, 12-oxo-10(*E*)-dodecenoic acid, or hexanal) per minute with alanine as amine donor. The formation of NADH is proportional to the transformation of the acceptor substrates as outlined in Fig. 1.

### Activity monitoring of the LOX, HPL, and ω-TA enzyme cascade

The product conversion in a one-pot reaction with HPL_CP-N_ and ω-TA was analyzed photometrically as described above (paragraph 2.6). Additionally, 10 µl of a 20 U ml^−1^ HPL_CP-N_ solution was added to the cuvette, and 0.1 mM 13*S*-HPODE was used as substrate. Reactions were started by the addition of NADH, and the decrease in the absorbance of NADH at 340 nm was monitored. Negative controls were performed by omitting the enzymes one by one to exclude a side reaction.

For a one-pot reaction with LOX-1, HPL_CP-N_, and ω-TA, the reaction mixtures were prepared in the same way. Additionally, 10 µl of 50 U ml^−1^ LOX-1 was added to the cuvette, and 0.1 mM linoleic acid was used as substrate. Again, reactions were started by the addition of NADH, and negative controls were performed by omitting the enzymes one by one.

### Biocatalytic synthesis of 12-aminododecenoic acid and hexylamine

Transaminase reactions of 500 µl were set up with 5 U ml^−1^ of purified ω-TA (unit activity based on coupled photometric assay with 12-oxo-9(*Z*)-dodecenoic acid substrate), 50 mM l-alanine and 0.1 mM pyridoxal-5-phosphate in 50 mM potassium phosphate buffer pH 7.5 containing 50 mM NaCl. Optionally, DMSO was added at concentrations from 5 to 20%. Reactions were started by the addition of either 2.5 mM 12-oxo-9(*Z*)-dodecenoic acid, 12-oxo-10(*E*)-dodecenoic acid or hexanal. Reactions were typically carried out at 22 °C for 1 to 5 h. Then, 900 µl of a 50:50 acetonitrile–water mixture was added to 100 µl of reaction solution to stop the reaction, and the mixtures were used for HPLC analysis.

One-pot reactions with 20 U ml^−1^ HPL_CP-N_ and 5 U ml^−1^ TR_AD_ were carried out in a volume of 500 µl. Reactions were run at 22 °C using 50 mM l-alanine, 0.1 mM pyridoxal-5-phosphate, and 1 mM or 2.5 mM 13*S*-HPODE as substrate in 50 mM potassium phosphate buffer pH 7.5 containing 500 mM NaCl. The enzyme reactions were conducted either simultaneously for 1 h or consecutively, with HPL_CP-N_ added at the beginning and incubated for 5 min before TR_AD_ was added for 1 h. In a third approach, TR_AD_ was added in the beginning and HPL_CP-N_ was added consecutively every 10 min for 1 h.

In addition, one-pot reactions with LOX-1, HPL_CP-N_, and TR_AD_ were conducted with 100 U ml^−1^ LOX-1, 20 U ml^−1^ HPL_CP-N_, and 5 U ml^−1^ TR_AD_ in a total volume of 500 µl. The reactions were carried out as described above with 1 mM or 2.5 mM linoleic acid as substrate. Three different reaction settings were tested. Either all enzymes were added in the beginning and incubated for 4 h or LOX-1 was preincubated with linoleic acid for 3 h before HPL_CP-N_ was added. After 5 min, TR_AD_ was added together with l-alanine and pyridoxal-5-phosphate and reacted for another hour. In a third approach, LOX-1 was preincubated with linoleic acid for 3 h before TR_AD_ was added to l-alanine and pyridoxal-5-phosphate, and HPL_CP-N_ was added in portions every 10 min over a period of 1 h.

### Analysis of products by liquid chromatography

Mass spectrometry data were obtained using an LC-30AD Nexera LC/MS system from Shimadzu (Kyoto, Japan) equipped with a Shimadzu SPD-M20A UV detector and Shimadzu LCMS-2020 mass spectrometry detector. Samples of 5 to 10 µl were loaded onto a Kromasil Orbit-100-C18 5 μm column (30 mm × 4.6 mm). Water (A) and acetonitrile (B) containing 0.1% formic acid were used as the mobile phase. A linear gradient was applied as follows: 0.1 min 20% B; 20% B to 90% B within 4 min; 1.1 min holding at 90% B. A flow rate of 1.0 ml min^−1^ was used. Samples were ionized by electron spray ionization (ESI) in negative and positive modes and recorded in the range of 50 to 700 m/z. For reference spectra, the standards 12-aminododecanoic acid, 12-oxo-9(*Z*)-dodecenoic acid, 12-oxo-10(*E*)-dodecenoic acid, hexanal, and hexylamine were used.

Quantification of 12-aminododecenoic acid was conducted using an LC-20AD XR Nexera Liquid Chromatograph from Shimadzu (Kyoto, Japan) equipped with an evaporative light scattering detector (ELSD) 100 (VWR, Radnor, PA, USA). Samples of 10 to 20 µl were loaded onto a LaChrom II + C18 RP column (250 mm × 4.6 mm, 5 µm particle size) from Hitachi (Chiyoda, Japan). As the mobile phase, water (A) and acetonitrile (B) containing 0.1% formic acid were used. A linear gradient was applied as follows: 20% B to 60% B within 14 min; 60% B to 80% B within 3 min; 80% B to 90% B within 5 min. A flow rate of 1.0 ml min^−1^ was used. Since 12-aminododecenoic acid is not commercially available, 12-aminododecanoic acid (Alfa Aesar, Haverhill, MA, USA) was used for calibration.

## Results

### Cloning and expression of ω-transaminases

ω-Transaminases (ω-TAs) have been successfully used for the amination of hydrophobic aldehydes including the synthesis of 12-aminododecanoic acid from the corresponding 12-oxododecanoic acid (Schrewe et al. 2013; Song et al. 2014). The structurally similar monounsaturated 12-oxododecenoic acid in its 9(*Z*)- or 10(*E*)-conformation can be obtained from linoleic acid by biocatalytic synthesis using the enzymes LOX and HPL with hexanal as a byproduct (Coenen et al. 2022). To elucidate whether these oxododecenoic acids can be used as substrates by ω-TAs, seven enzyme candidates were evaluated. As a starting point, we selected the transaminase from *C. violaceum* (TR_CV_) with proven activity towards 12-oxododecanoic acid. In addition, we chose the transaminase from *P. denitrificans* (TR_PD_) with a sequence identity of 38.31% to the *C.* *violaceum* enzyme (Table S1). This enzyme was reported to transform 6-oxohexanoic acid to the corresponding 6-aminohexanoic acid (Sattler et al. 2014). The transaminases TR_2_ from *Acidihalobacter* sp*.* as well as TR_3_ and TR_6_ from uncultured *Rhodobacteraceae* bacteria have been shown to convert bulky ketones and hexanal (Coscolín et al. 2019) and were also included in the enzyme screening. They share sequence identities of 58.94%, 34.54%, and 54.75%, respectively, to TR_CV_. In addition, a BLAST search was performed to identify additional homologues of TR_CV_. Transaminases from *A. denitrificans* (TR_AD_) and *S. delicatus* (TR_SD_) were selected with identities of 81.05% and 53.64% with respect to TR_CV_ (Table S1).

**Fig. 2:**
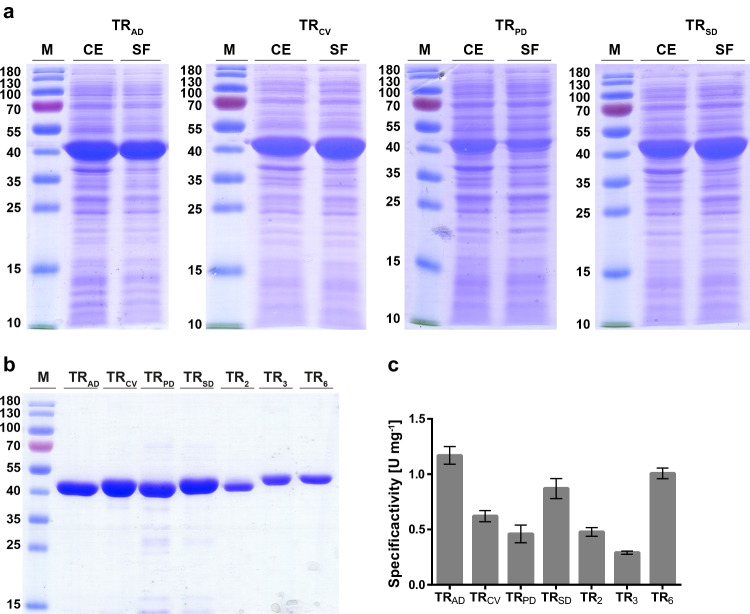
Purification and characterization of transaminases. **a** SDS-PAGE analysis after overexpression of transaminases TR_AD_, TR_CV_, TR_PD_, and TR_SD_ with CE = crude extract, SF = soluble fraction and M = marker protein ladder. **b** SDS-PAGE of eluate fractions of transaminases after purification with metal affinity chromatography. **c** Specific activities of the purified ω-TAs with hexanal as substrate in U mg^−^.^1^ with 1 Unit (U)

Gene sequences encoding TR_CV_, TR_AD_, TR_PD_, and TR_SD_ were codon-optimized for expression in *E. coli* and synthesized by BioCat GmbH (Heidelberg, Germany). They were cloned into the pET-21(b) + expression vector, and *E coli* BL21(DE3) was transformed with the respective vectors. The recombinant strains were grown in TB medium containing ampicillin at 20 °C for 24 h. Overexpression of the transaminases was analyzed with SDS-PAGE (Fig. 2a). Strong protein bands representing TR_CV_, TR_AD_, TR_PD_, and TR_SD_ were visible in both the crude extracts (CE) and, after separation of cell fragments, in the soluble fractions (SF). All transaminases were His6-labelled and successfully purified by metal affinity chromatography (Fig. 2b). Proteins were eluted, and minor impurities were observed only in the TR_PD_ and TR_SD_ fractions. Overexpression of TR_CV_ and TR_AD_ resulted in total yields of 41 mg for TR_AD_ and 80 mg for TR_CV_ were obtained from 50 ml of culture broth. For TR_PD_ and TR_SD_, the yields were lower; nevertheless, 13 and 17 mg of purified enzymes were obtained from 50 ml culture. Transaminases TR_2_, TR_3_, and TR_6_ were expressed in *E. coli* MC1061 as described previously (Coscolín et al. 2019). The protein masses of 45–55 kDa observed in SDS‒PAGE corresponded well with the calculated masses of the His6-tagged ω-TAs, which ranged from 48.42 to 51.25 kDa. The highest protein concentrations were measured in the eluate fractions of TR_CV_ at 13.9 mg ml^−1^, followed by TR_3_ and TR_AD_ at 5.21 mg ml^−1^ and 5.14 mg ml^−1^, respectively.

Functional expression of the ω-TAs was analyzed with hexanal as a reference substrate (Fig. 2c). For this purpose, a coupled enzymatic activity assay was established with l-alanine, lactate dehydrogenase, and NADH (Fig. 1). The specific transaminase activities were calculated from the initial decrease in NADH. Negative controls in which enzymes, substrate or cofactor were sequentially omitted showed only minor background reactions, thus confirming the functionality of the enzyme assay. Figure 2c shows that all enzymes were functionally expressed and accepted hexanal as a substrate, with TR_AD_ exhibiting the highest specific activity with 1.17 U mg^−1^, followed by TR_6_ and TR_SD_.

### Transaminase activity analysis in a coupled enzyme system

**Fig. 3:**
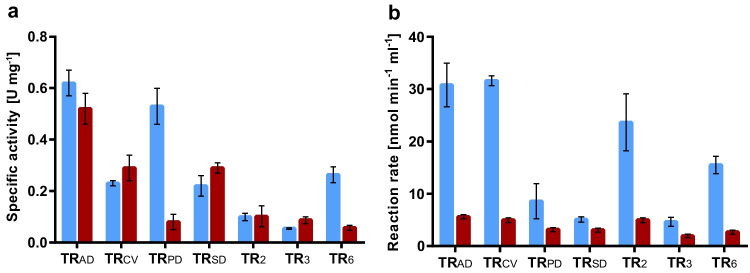
Substrates conversion of transaminases. **a** Specific activity of ω-TAs against different aldehyde substrates determined with a coupled photometrical enzyme assay with lactate dehydrogenase (LDH) and NADH at 340 nm. Blue: 12-oxo-9(*Z*)-dodecenoic acid and red: 12-oxo-10(*E*)-dodecenoic acid. **b** Conversion rate of NADH to NAD^+^ catalyzed by an enzyme cascade containing the ω-TAs in combination with HPL_CP-N_ (blue bars) and in combination with HPL_CP-N_ and LOX-1 (red bars) in a coupled photometrical enzyme assay with LDH and NADH

12-Oxo-9(*Z*)-dodecenoic acid and 12-oxo-10(*E*)-dodecenoic acid were tested as ω-TA substrates in the coupled photometric assay. All seven enzymes accepted both oxoacids, revealing a broad substrate spectrum of the ω-TAs (Fig. 3a). In general, the shorter-chain aldehyde hexanal (Fig. 2c) was the best substrate for the ω-TAs except for TR_PD_, which exhibited higher specific activity for 12-oxo-9(*Z*)-dodecenoic acid. TR_AD_ showed the highest specific activities, with 0.62 U mg^−1^ for 12-oxo-9(*Z*)-dodecenoic acid and 0.52 U mg^−1^ for 12-oxo-10(*E*)-dodecenoic acid. Although the double bonds are in direct proximity to the oxo-group, their position does not seem to affect their acceptance as transaminase substrates. Remarkably, five of the seven transaminases transformed the 10(*E*)- and 9(*Z*)-isomers with comparable activity. Especially in the 10(*E*)-configuration, a stabilized double bond system is formed in conjugation with the oxo-group, which should be a demanding substrate for transaminases. However, only TR_PD_ and TR_6_ exhibited significantly lower activities towards the 10(*E*) isomer.

Coupling of transaminases with enzymes from the oxylipin pathway reveals a route for the synthesis of polyamide precursors from linoleic acid. To elucidate the feasibility of this pathway, we first combined ω-TAs with HPL_CP-N_ and monitored the NADH decrease in the LDH coupled photometric assay. The initial overall activity could be monitored as the sole parameter because hexanal and oxododecenoic acid are measured simultaneously. 13*S-*HPODE, which was synthesized with LOX-1 as described previously (Gala Marti et al. 2021), was used as substrate, and purified papaya HPL_CP-N_ expressed in *E. coli* (Coenen et al. 2022) was applied in the enzyme assay. Enzymatic activity was demonstrated for all seven ω-TAs (Fig. 3b). The highest conversion rates of 31.6 nmol min^−1^ ml^−1^ were obtained with TR_CV_, followed by TR_AD_ and TR_2_. TR_AD_ and TR_CV_ already showed relatively high specific activities in the single substrate measurements (Fig. 3a), whereas TR_2_ exhibited relatively low specific activities. In contrast, TR_SD_ performed better in the single-substrate measurements than in the enzyme cascade with HPL_CP-N_. Transaminase deactivation by the reactive hydroperoxide substrate 13*S*-HPODE or differences in transaminase substrate affinities may explain these differences.

Next, one-pot enzyme reactions were performed using soybean LOX-1, HPL_CP-N_ and ω-TAs with linoleic acid as the substrate. Again, coupling to LDH and monitoring of NADH decrease was used for analysis of initial activities. A positive reaction was confirmed with all transaminases, although the overall activity was lower than that in the HPL–ω-TA system (Fig. 3b). The highest activity of 5.6 nmol min^−1^ ml^−1^ was measured with TR_AD_, followed by TR_CV_ and TR_2_ exhibiting 4.9 nmol min^−1^ ml^−1^ each.

### Development of an enzyme cascade with transaminase TRAD

**Fig. 4:**
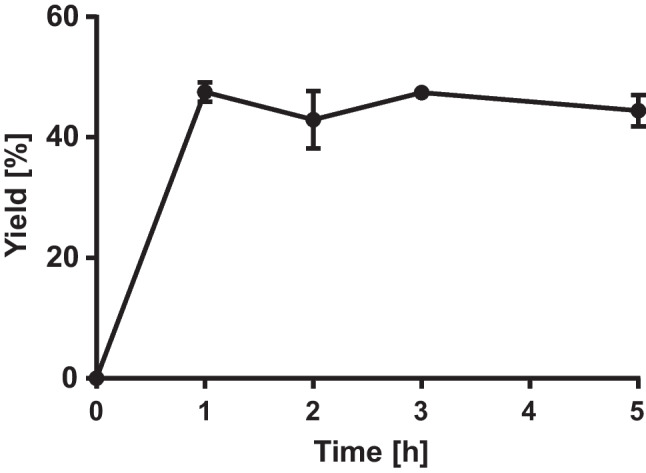
Time-dependent formation of 12-aminododecenoic acid according to LC-ELSD analysis

TR_AD_ was selected for the further development of a coupled enzyme cascade with oxylipin pathway enzymes. To monitor the synthesis of 12-aminododecenoic acid and hexylamine individually, the establishment of an analytical method was needed. For the detection of amino dodecenoic acids, we developed an LC-based analysis using ELSD detection (Fig. 4). Quantification of 12-aminododecenoic acid was possible using commercially available 12-aminododecanoic acid as a reference standard for the ELSD detector (Figure S1a). The formation of 12-aminododecenoic acid with TR_AD_ was monitored over a period of 5 h. A maximum conversion of 47% was obtained at a substrate concentration of 2.5 mM within 1 h (Fig. 4). Hence, a 1-h reaction time was chosen for the development of the enzyme cascade.

For product verification, LC was coupled with a mass spectrometer. The mass spectrum of 12-aminododecenoic acid reveals a major peak at 214 m/z, which correlates to the molecular weight of its protonated form (Fig. S1b). Comparative analysis of 12-aminododecanoic acid possessing two extra hydrogen atoms gave a mass spectrum with a major peak at 216 m/z (Figure S1c). Transamination of 12-oxo-9(*Z*)- and 12-oxo-10(*E*)-dodecenoic acid (Figure S2a) resulted in similar mass spectra with the same peak at 214 m/z in each case (Figure S1d). The transformation of hexanal to hexylamine was monitored accordingly, and the mass spectrum of the TR_AD_ reaction product correlated with the mass spectrum of a hexylamine reference exhibiting a peak of 102 m/z for the protonated molecule (Figure S2 b, c).

**Fig. 5:**
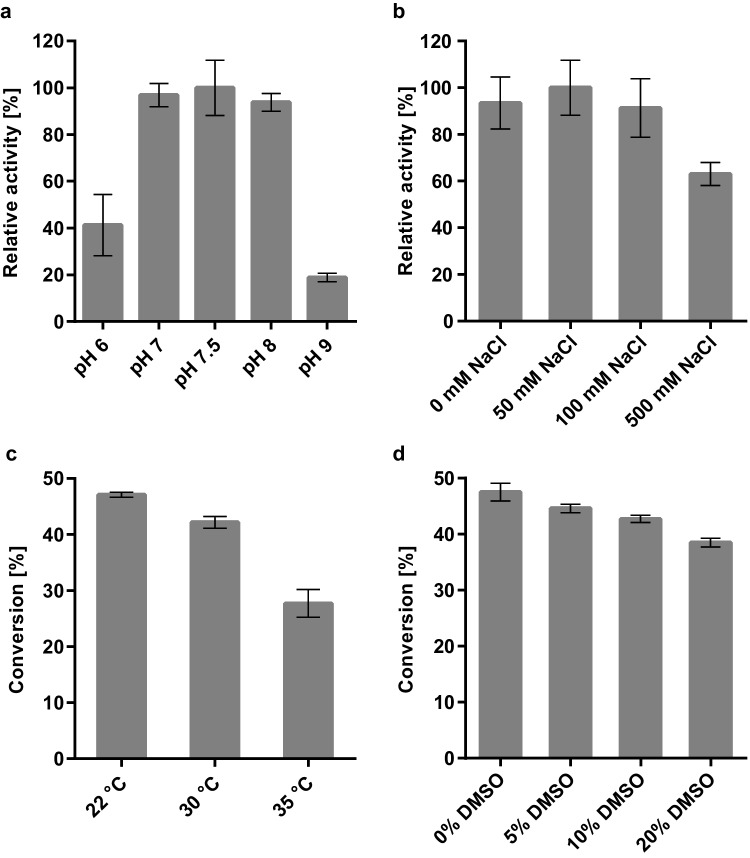
Analysis of reaction conditions for TR_AD_ with monitoring of **a** pH, **b** salt concentration, **c** reaction temperature and **d** addition of DMSO. **a**, **b** pH and salt optima were analyzed photometrically with LDH and NADH coupling using 12-oxo-9(*Z*)-dodecenoic acid as substrate. The highest relative activities were set to 100% (for pH 7.5 1.7 U ml^−1^ and for 50 mM NaCl 1.8 U ml^−1^). **c**, **d** Temperature and DMSO optima were analyzed in biocatalytic reactions with LC-ELSD quantification

TR_AD_ exhibited a pH optimum of 7.5 with remaining activities of approximately 40% at pH 6 and 20% at pH 9 (Fig. 5a). While LOX-1 has its pH optimum at approximately pH 9, HPL_CP-N_ performs best at pH 6 (Coenen et al. 2022). Since our previous study showed sufficient activity of LOX-1 and HPL_CP-N_ in a one-pot reaction at pH 7.5, this pH was expected to be suited for the three-enzyme cascade. The optimum NaCl concentration of TR_AD_ was 50 mM, and more than 60% activity was maintained at high salt concentrations of 500 mM (Fig. 5b). HPL_CP-N_ requires high salt concentrations for optimal activity (Coenen et al. 2022); therefore, a salt concentration of 500 mM NaCl was chosen for the enzyme cascade. LOX-1 and HPL_CP-N_ are temperature-sensitive enzymes, and thus, a low reaction temperature should be applied in the enzyme cascade reaction. The highest 12-aminododecenoic acid conversion by TR_AD_ was achieved at room temperature (22 °C) (Fig. 5c) and was chosen for all further experiments. It has been reported that transaminases, including TR_2_, TR_3_, and TR_6_, exhibit higher activities in the presence of DMSO (Coscolín et al. 2019). In contrast, we did not observe an activating effect of DMSO on TR_AD_ (Fig. 5d); thus, the enzyme cascade was established under solvent-free conditions.

**Fig. 6:**
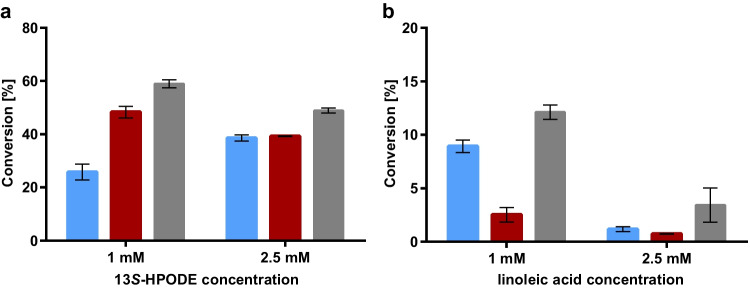
One-pot reactions of oxylipin pathway enzymes with ω-transaminase TR_AD_ for the synthesis of 12-aminododecenoic acid. **a** HPL_CP-N_ was coupled with TR_AD_ using 1 mM and 2.5 mM 13*S*-HPODE as substrate. Blue bars = simultaneous enzyme addition, red bars = HPL addition before TR_AD_, grey bars = TR_AD_ addition before HPL dosage in portions. **b** LOX-1 was coupled with HPL_CP-N_ and TR_AD_ using 1 mM and 2.5 mM linoleic acid as substrate. Blue bars = simultaneous enzyme addition, red bars = LOX-1 was added before HPL_CP-N_ and then TR_AD_ was added, grey bars = LOX-1 was added before TR_AD_ addition and HPL_CP-N_ dosage in portions

During the development of an enzyme cascade for the transformation of safflower oil to oxododecenoic acid, we discovered that the consecutive addition of enzymes resulted in better substrate conversion (Coenen et al. 2022). Therefore, simultaneous and consecutive enzyme addition was tested to determine the best conditions for a coupled HPL_CP-N_–TR_AD_ reaction. Two modes of consecutive enzyme additions were analyzed. Either HPL_CP-N_ was incubated with 13*S*-HPODE as substrate for 5 min before TR_AD_ was added or TR_AD_ was added at the beginning of the reaction, and HPL_CP-N_ was dosed in 6 portions and added every 10 min for 1 h. The highest 12-aminododecenoic acid conversion of 59% was obtained with stepwise HPL addition at a substrate concentration of 1 mM (Fig. 6a). This setup prevents a too-rapid synthesis of 12-oxo-9(*Z*)-dodecenoic acid, which is unstable in the presence of high protein concentrations (Coenen et al. 2022). Thus, if 12-oxo-9(*Z*)-dodecenoic acid is formed stepwise, TR_AD_ has enough time for the conversion to 12-aminododecenoic acid before 12-oxo-9(*Z*)-dodecenoic acid degradation. The conversion of 13*S*-HPODE with stepwise HPL addition was comparable to the reaction of 12-oxododecenoic acid at a substrate concentration of 2.5 mM with TR_AD_ alone. Hence, this two-enzyme system performs equally well.

The three-enzyme cascade with LOX-1, HPL_CP-N_, and TR_AD_ was analyzed in a similar way with both simultaneous and consecutive enzyme addition of HPL and ω-TA. According to the results of Gala Marti et al. (2021), the LOX-1 reaction proceeds over a period of 3–5 h under the chosen reaction conditions. Therefore, reactions were started with a 3-h preincubation with LOX-1 and linoleic acid as substrates. Then, HPL_CP-N_ was applied 5 min before TR_AD_ addition or TR_AD_ was added first, and HPL_CP-N_ was dosed in portions every 10 min for 1 h. The highest conversion of 12-aminododecenoic acid was again obtained in the reaction setup with stepwise HPL addition. The maximum conversion was lower than that in the 2-enzyme system and reached 12%; however, the general feasibility of a three-enzyme cascade with LOX-1, HPL and ω-TA was demonstrated (Fig. 6b).

## Discussion

The vast majority of polymer production still relies on petroleum-based naphtha as a raw material. In recent years, several approaches have been developed for the synthesis of biologically based polymer precursors (Chung et al. 2015). 12-Aminododecanoic acid, the monomer for nylon-12 production, was synthesized from vegetable oil based raw materials with an engineered *E. coli* biocatalyst (Schrewe et al. 2013; Ladkau et al. 2016). However, this biocatalytic route uses lauric acid as the starting material, which can only be obtained in large quantities from palm kernel and coconut oil. Both coconut and oil palms can grow in wet tropical climates only, and an increase in oil production will increase pressure on pristine rainforests, leading to ecological problems (Dislich et al. 2017; Qaim et al. 2020). To circumvent this issue, we developed an alternative enzymatic route towards nylon-12 monomers, which uses linoleic acid as the starting material. Linoleic acid-rich oils are available from safflower and sunflower, and large-scale plantations are found in temperate and subtropical climate zones.

The ω-transaminases analyzed in our study originated from three different bacterial families: the *Rhodobacteraceae* family comprising TR_PD_, TR_SD_, TR_3_, and TR_6_, the *Chromobacteriaceae* family comprising TR_CV_ and TR_AD,_ and the *Ectothiorhodospiraceae* family comprising TR_2_. Differences between *P.* *denitrificans* and *C.* *violaceum* ω-TA become visible when analyzing the available crystal structures (Humble et al. 2012; Rausch et al. 2013). Interestingly, three residues in the active sites of the substrate pockets differ. While TR_CV_ harbors residues M56, M166, and C418, TR_PD_ harbors N53, K163, and L417 in the corresponding positions. These changes have been described to result in larger and more hydrophobic active sites in TR_CV_, which could lead, for example, in higher activity towards large, hydrophobic compounds such as long-chain aliphatic substrates (Rausch et al. 2013). In total, 17 amino acids were identified and designated to be important for substrate binding: four inside the S- (small) pocket and 12 inside the L- (large) pocket. Additionally, K285 (numeration of TR_PD_) was shown to bind the cofactor PLP. TR_AD_ is the only transaminase that shares all 17 amino acid residues with TR_CV_ (Figure S3), suggesting a similar structure of the substrate pockets. In contrast, the other ω-TAs share only 14 (TR_SD_, TR_2_, and TR_6_, TR_PD_) or 10 (TR_3_) of these amino acids, potentially leading to some structural variations. In accordance, phylogenetic analysis revealed that TR_AD_ is most closely related to TR_CV_, whereas TR_SD_, TR_2_, and TR_6_ are significantly more distant, with sequence identities of approximately 55% (Figure S4, Table S1). TR_PD_ and TR_3_ are the least homologous transaminases, with sequence identities of approximately 35% to TR_CV_ and only 32% between each other.

Interestingly, despite differences in the sequences and amino acid architecture of their substrate pockets, all ω-TAs accepted unsaturated long-chain oxoacids as substrates. A clear substrate preference could not be deduced from the comparison of the three substrates tested. Most ω-TAs preferred hexanal, indicating that unsaturated oxoacids are more structurally challenging transaminase substrates. The only exception was TR_PD_, which showed higher specific activity for 12-oxo-9(*Z*)-dodecenoic acid than for hexanal. Additionally, the enzyme had a significantly lower activity with the 10(*E*) isomer, whereas the other transaminases showed less differentiation between the two isomers. In the coupled enzyme assay with HPL_CP-N_, a clearer distinction between the ω-TA groups was possible. Here, TR_CV_ and TR_AD_ showed the highest initial activities. Hexanal and ω-oxododecenoic acid are formed in situ by HPL_CP-N_, and thus, their substrate concentration is initially low. Therefore, TR_CV_ and TR_AD_ must possess higher substrate affinities than the other transaminases. The more hydrophobic active sites of these two transaminases may allow better substrate binding, making these enzymes better suited for the development of an enzyme cascade.

Coupling TR_AD_ with HPL_CP-N_ resulted in a similar substrate conversion as using TR_AD_ alone. Thus, at substrate concentrations of 2.5 mM, the functionality of the two-enzyme cascade was proven. However, the three-enzyme cascade with LOX-1 resulted in a much lower conversion. In our previous study, we showed that the HPL reaction is significantly faster than the LOX reaction (Coenen et al. 2022). Hence, the slow formation of 13*S*-HPODE could be the cause of the lower overall activity observed in the coupled three-enzyme system. To circumvent this problem, reaction setups were tested, in which LOX-1 and linoleic acid were preincubated for 3 h before TR_AD_ was added, and HPL_CP-N_ was added in portions over an hour. Indeed, this reaction setup gave higher product concentrations than the simultaneous enzyme addition. Nevertheless, product concentrations of 12% and 3.5% at substrate concentrations of 1 mM and 2.5 mM, respectively, demand further reaction optimization. Low yields in a coupled LOX and HPL system have also been observed previously for the synthesis of 9-oxononanoic acid (Otte et al. 2013). Here, yields decreased significantly with increasing substrate concentration, and in accordance with our findings, the product conversion was significantly higher when LOX was preincubated with the substrate prior to the addition of HPL.

Another issue in the development of a LOX–HPL–ω-TA enzyme cascade is the low stability of 12-oxo-9(*Z*)-dodecenoic acid. Rapid isomerization to 12-oxo-10(*E*)-dodecenoic acid was observed within few minutes and was presumed to be caused either by keto-enol tautomerism or by Schiff base formation in the presence of high protein concentrations (Coenen et al. 2022). The development of a whole-cell biocatalyst for the continuous production of 12-aminododecenoic acid may overcome the aforementioned problems by adjusting the respective enzyme activities. Whole-cell biocatalysts have already been established with LOX and HPL (Buchhaupt et al. 2012; Otte et al. 2014); hence, incorporation of ω-TA should be feasible. In addition, a regeneration system for l-alanine and PLP could be engineered, which may be important for the conversion of higher substrate concentrations. In particular, an imbalance in alanine–pyruvate concentration can lead to an undesired shift of ω-TA-catalyzed alanine formation. Ge et al. (2020) established a whole-cell biocatalyst for the synthesis of 12-aminododecanoic acid from dodecanoic acid by enzymatic ω-hydroxylation, oxidation, and ω-amination in combination with a regeneration system for the cosubstrate l-alanine and the cofactors NADPH, NADH, and PLP and achieved a yield of 96.5% at a concentration of 5 mM. For cofactor regeneration, the endogenous cell pathways were used in combination with exogenous ribose 5-phosphate (R5P)-dependent PLP synthesis. A similar enzyme cascade with ω-TA and cofactor regeneration was developed by Kim et al. (2020), yielding up to 77.3% 11-aminoundecanoic acid from ricinoleic acid at substrate concentrations of 300 mM. These experiments indicate that an engineered whole-cell biocatalyst with a cofactor regeneration system may be suitable for a coupled LOX–HPL–ω-TA enzyme cascade.

It has to be mentioned that polymerization of our reaction product 12-amino-9(*Z*)-dodecenoic acid will result in unsaturated polyamide 12. Unsaturated polyamides were reported more than 80 years ago and the double bond is useful for polymer crosslinking (Pryde 1979). Several biobased unsaturated polyamides have been described more recently and were proposed for example as thermoreactive sealants (Radzik et al. 2020). To obtain saturated polyamide 12 hydrogenation of the double bond is needed. Standard chemical methods like hydrogenation with molecular hydrogen using palladium on coal catalysts may be applied (Mudiyanselage et al. 2014). Alternatively, ene-reductases may be an interesting biocatalytic option for double bond hydrogenation especially for the design of a whole cell biocatalyst. Ene-reductases exhibit broad substrate spectra and are currently applied for the asymmetric reduction of C–C double bonds (Toogood and Scrutton 2018).

In summary, seven ω-TAs were successfully cloned, expressed and purified, and their activity towards long-chain aldehydes was verified. TR_AD_ from *A. denitrificans* was selected for the development of a three-enzyme cascade with soybean LOX-1 and papaya HPL_CP-N_. Starting from safflower oil-based linoleic acid, the synthesis of 12-aminododecenoic acid, a precursor for the synthesis of the biobased polyamide nylon-12, was demonstrated.

## Supplementary Information

Below is the link to the electronic supplementary material.

Supplementary file1 (PDF 435 kb)

## Data Availability

Data are available on request.
