# Supplementary material for: Synthesis of 12-aminododecenoic acid by coupling transaminase to oxylipin pathway enzymes
Source: Appl Microbiol Biotechnol. 2023 Feb 21;107(7-8):2209–21. doi: 10.1007/s00253-023-12422-6 (PMC10033567; doi:10.1007/s00253-023-12422-6)
Supplement: Supplementary file 1 — Supplementary file1 (PDF 435 kb) [file 253_2023_12422_MOESM1_ESM.pdf]

Supplementary data:

Synthesis of 12-aminododecenoic acid by coupling transaminase to oxylipin pathway enzymes

Anna Coenen[1], Manuel Ferrer[2], Karl-Erich Jaeger[3,4], Ulrich Schörken[1*]

[1]TH Köln – Campus Leverkusen, Faculty for Applied Natural Sciences, Leverkusen, Germany

[2]ICP, CSIC, Madrid, Spain

[3]Institute of Molecular Enzyme Technology, Heinrich Heine University Düsseldorf, Forschungszentrum Jülich, Jülich, Germany

[4]Institute of Bio- and Geosciences IBG-1: Biotechnology, Forschungszentrum Jülich GmbH, Jülich, Germany

* Correspondence: ulrich.schoerken@th-koeln.de; +49-214-32831-4610

Table S1 Sequence identity of transaminases with respect to each other, calculated with BLAST

	**TR_{AD}**	**TR_{CV}**	**TR_{PD}**	**TR_{SD}**	**TR_2**	**TR_3**	**TR_6**
**TR_{AD}**	-	81.05	36.32	54.78	56.73	35.31	56.43
**TR_{CV}**	81.05	-	38.31	53.64	58.94	34.54	54.75
**TR_{PD}**	36.32	38.31	-	34.15	35.64	32.05	35.49
**TR_{SD}**	54.78	53.64	34.15	-	58.65	35.02	69.05
**TR_2**	56.73	58.94	35.64	58.65	-	36.93	60.05
**TR_3**	35.31	34.54	32.05	35.02	36.93	-	34.36
**TR_6**	56.43	54.75	35.49	69.05	60.05	34.36	-

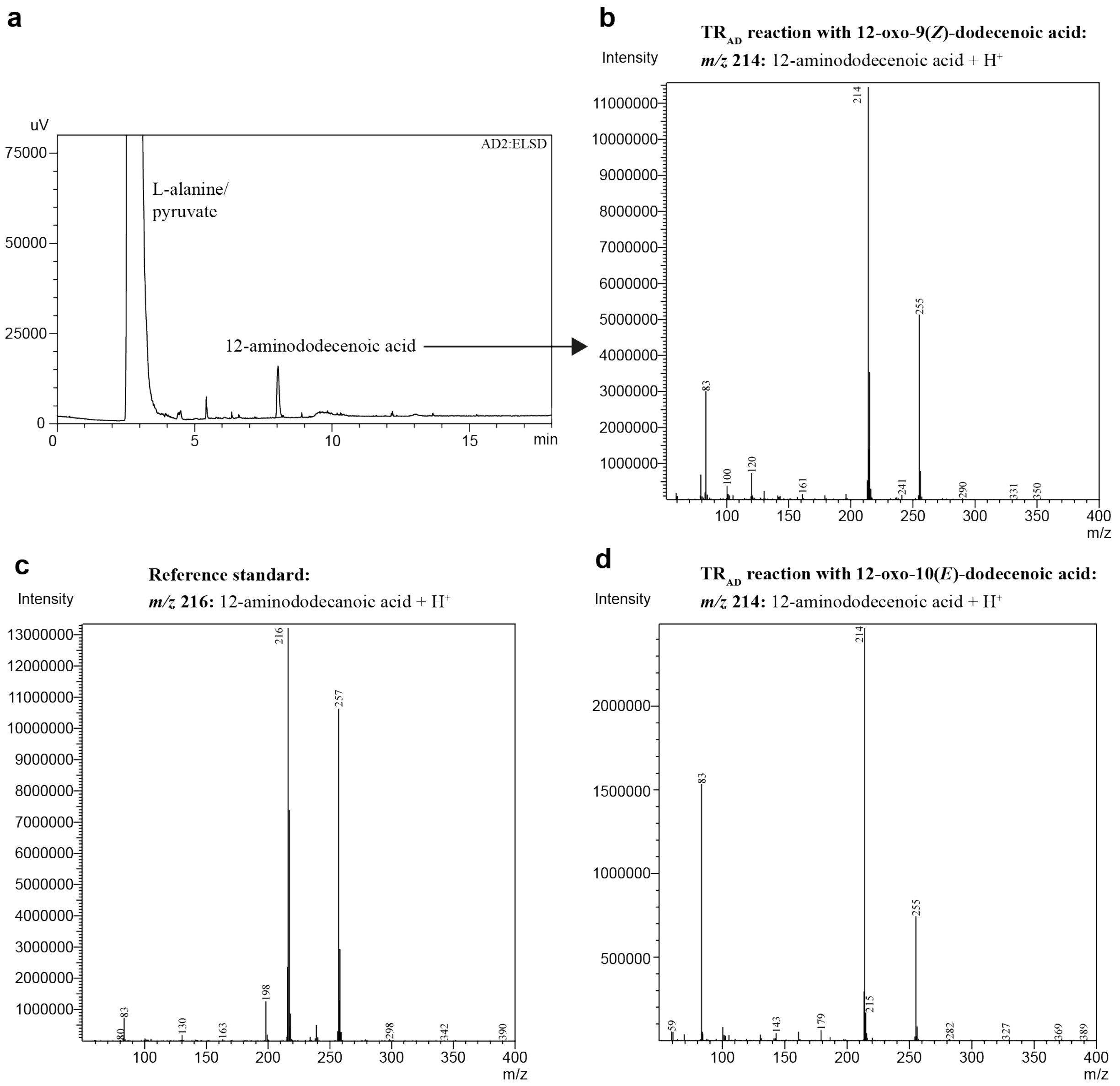

Fig. S1 Analysis of reaction products of TR_{AD} enzyme reaction. (**a**) ELSD chromatogram of the TR_{AD} reaction product from the conversion of 12-oxo-9(*Z*)-dodecenoic acid. Mass spectra of 12-aminododecenoic acid after TR_{AD} reaction with 12-oxo-9(*Z*)-dodecenoic acid (**b**), standard reference 12-aminododecanoic acid (**c**) and 12-aminododecenoic acid after TR_{AD} reaction with 12-oxo-10(*E*)-dodecenoic acid (**d**)

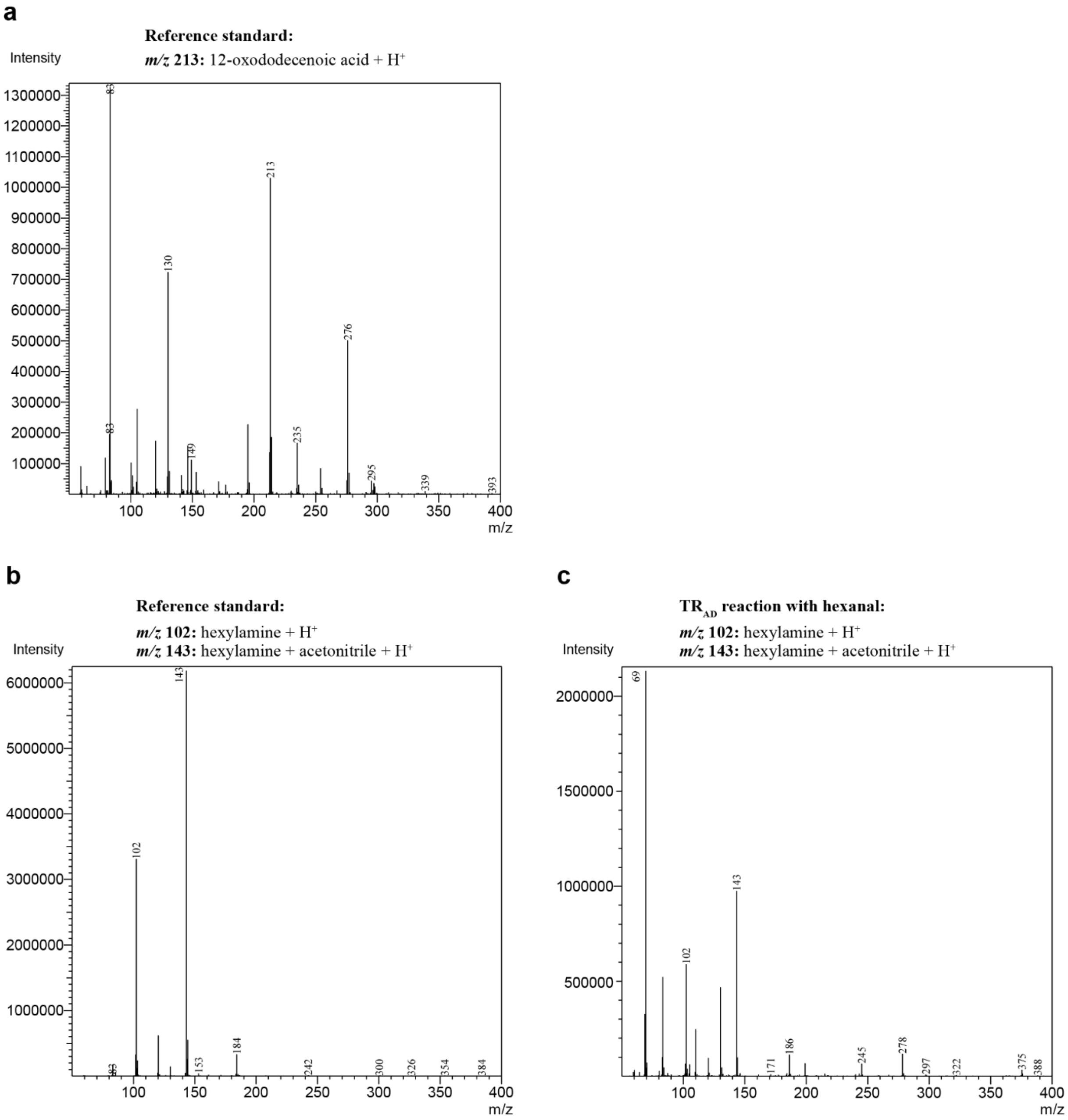

Fig. S2 Mass spectra from LC-MS analysis of 12-oxododecenoic acid reference standard (**a**), hexylamine reference standard (**b**) and hexylamine formation after TR_{AD} reaction with hexanal (**c**)

```
TR3       ------MKDENFLKENNARHLWHPMGAPGDLQANTPKIITGASGVSITDIDGHQTVDAVG    54
TRPD      ------MNQPQSWEARAETYSLYGFTDMPSVHQRGTVVVTHGEGPYIVDVHGRRYLDANS    54
TRSD      MPSITNHLPTAELQALDSAHHMHPFTTNDELTQKGARVITRAKGIYLTDSEGNEILDAMA    60
TR6       MVQITNHMPTAELQALDAAHHMHPFTTQSELAERGARVITRAEGAYIYDSEGNKILDGMA    60
TR2       --MSQSQRSTADWQRLDAAHHLHPFTDYGELNTKGSRIITRAEGCYLWDSDGNQILDGMA    58
TRCV      ---MQKQRTTSQWRELDAAHHLHPFTDTASLNQAGARVMTRGEGVYLWDSEGNKIIDGMA    57
TRAD      ---MQNQRTTTEWRELDAAHHLHPFTDTNSLNQQGARVITKADGIYLYDSEGNKILDGMA    57

TR3       GLWCVNLGYSNDVVKEAIAKQLYDLPYYSAFAGTSNPPAIEASYAVREFFAEDGMGRVFF    114
TRPD      GLWNMVAGFDHKGLIEAAKAQYDRFPGYHAFFGRMSDQTVMLSEKLVEV-SPFDNGRVFY    113
TRSD      GLWCVNLGYGREEMGQVAARQMNELPYYNTFFQTTHVPAIALAKELADL-APGDLNYVFF    119
TR6       GLWCVNIGYGRQELVDVAARQMAELPYYNTFFMTTHVPAIALSAKLAEL-APAHLNHVFY    119
TR2       GLWCVNIGYGRKELAEVAYRQMQELPYYNNFFQCSHPPAIELSRLLSEV-TPKHMNHVFF    117
TRCV      GLWCVNVGYGRKDFAEAARRQMEELPFYNTFFKTTHPAVVELSSLLAEV-TPAGFDRVFY    116
TRAD      GLWCVNIGYGRKDLPEVAKQQMEQLAYYNTFFKTTHPAVVELSHLLAEV-APEGFKQVFY    116

TR3       TSGGSDSVETALRLARQYHRLRGEPTRTKYISLKKGYHGTHFGGASVNGNNRFRINYEPL    174
TRPD      TNSGSEANDTMVKMLWFLHAAEGKPQKRKILTRWNAYHGVTAVSASMTGKPYN-SVFGLP    172
TRSD      AGSGSEANDTNLRMVRTYWAQKGKPEKSHVISRKNAYHGSSVGSASLGGMTPMHEQGGLP    179
TR6       SSSGSEANDTNIRLVRTYWAEKGKPSKSIIISRHNAYHGSTLGGASLGGMGGMHAQGGLP    179
TR2       TGSGSDSNDTILRMVRYYWKLLGKPYKKVVISRENAYHGSTVAGASLSGMKAMHAQGDLP    177
TRCV      TNSGSESVDTMIRMVRRYWDVQGKPEKKTLIGRWNGYHGSTIGGASLGGMKYMHEQGDLP    176
TRAD      TNSGSESVDTMIRMVRRYWDVKGKKDKKTLIGRWNGYHGSTIGGASLGGMTYMHEQGDLP    176

TR3       LPGCFHLPSPYPYRNPFNETDPAQLAQNIAAAFEDEIAFQDANTIAAFIMEPIQGAGGVI    234
TRPD      LPGFIHLTCPHYWRYGEEGETEAQFVARLARELEDTITREGADTIAGFFAEPVMGAGGVI    232
TRSD      IPGIHHIGQPDWWAEGGDQ-SPEEFGLARARELEDKILELGADNVAAFIGEPIQGAGGVV    238
TR6       IPDIHHIDQPNWWAEGGDM-DPAEFGLERAQQLEKAILKLGEDRVAAFIAEPVQGAGGVI    238
TR2       IPGIEHIEQPYHFGRAPDM-DPAEFGRQAAQALERKIDEIGECNVAAFIAEPIQGAGGVI    236
TRCV      IPGMAHIEQPWWYKHGKDM-TPDEFGVVAARWLEEKILEIGADKVAAFVGEPIQGAGGVI    235
TRAD      IPGIVHVEQPWWYKHGKDM-TPEEFGLAAAKWVEDKILEVGADKVAAFVGEPIQGAGGVI    235

TR3       VPDASFMGLMRDICDRHGILLISDEVITGFGRTGDWSGARHWGVKPDLMTTAKGITSGYF    294
TRPD      PPAKGYFQAILPILRKYDIPMISDEVICGFGRTGNTWGCLTYDFMPDAIISSKNLTAGFF    292
TRSD      IPPSTYWPEIQRICDKHDVLLIADEVICGFGRTGNWFGSQTMGIKPHIMTIAKGLSSGYA    298
TR6       VPPETYWPEIQRICDKYEILLIADEVICGFGRTGNWFGSETVGWKPDIMTIAKGLSSGYQ    298
TR2       IPPDSYWPEIKRICAERDILLIVDEVITGFGRLGTWFGSQYYDLQPDLMPIAKGLSSGYM    296
TRCV      VPPATYWPEIERICRKYDVLLVADEVICGFGRTGEWFGHQHFGFQPDLFTAAKGLSSGYL    295
TRAD      VPPSTYWPEIQRICQKYDILLVADEVICGFGRTGEWFGQQVFGFKPDIFTTAKGLSSGYQ    295

TR3       PVGACLLSEAVAEVFEKDTSGEAAIYHGYTYSAHPVGAAAVVATLAETQRLDLKTNAAA-    353
TRPD      PMGAVILGPDLAKRVEAAVEAIEEFPHGFTASGHPVGCAIALKAIDVVMNEGLAENVRRL    352
TRSD      PIGGSIVCDEVAEVI-----NACEFNHGYTYSGHPVCAAVALENLRIMQEENIIDHVQNV    353
TR6       PIGGSIVSDEIATVI-----GNCEFNHGYTYHAHPVAAAVALENLRILDEEGIVARVRDE    353
TR2       PIGGVMVSDRVAKVV---IEEGGEFFHGYTYSGHPVAAAVAAENIRIMRDEGIIERAGAE    353
TRCV      PIGAVFVGKRVAEGL----IAGGDFNHGFTYSGHPVCAAVAHANVAALRDEGIVQRVKDD    351
TRAD      PIGAVFVNEKVATTL----AEGGDFNHGFTYSGHPVAAAVAHANVKALRDEGIVDRVKND    351

TR3       RGTQLFEGVKKLAEKHDIIGDVRGGHGLMTGIEIVSDKAAKTPMDNET---MKRIHQTAY    410
TRPD      -APRFEAGLKRI-ADRPNIGEYRG-IGFMWALEAVKDKPTKTPFDANLSVS-ERIANTCT    408
TRSD      AAPALQEALNKL-GEHPLVGGVNV-SGLMASLPLTPHKESRAKFASDAGTAGYLCREHCF    411
TR6       TGPYLAQKWAAM-ADHPMVGEASI-VGMMGSIALTPNKSTRATFKAEAGTVGYICRERCF    411
TR2       IAPYLQARWREL-GEHPLVGEARG-VGMVAALELVKSKQPLERFE-EPGKVGSLCRDLSV    410
TRCV      IGPYMQKRWRETFSRFEHVDDVRG-VGMVQAFTLVKNKAKRELFP-DFGEIGTLCRDIFF    409
TRAD      TGPYMQKRWREVFGQFEHVDDVRG-VGLIQAFTLVKNKATRELFP-NFGEIGTMCRDIFF    409

TR3       EAGAMVRLGAHNVLMSPPLTISEAEVNTILTALDAGFSAA--------------    450
TRPD      DLGLICRPLGQSIVLCPPFILTEAQMDEMFEKLEKALDKVFAEVA---------    453
TRSD      ANNLVMRHVGDRMIISPPLIITPEEIAIFADRATRALDATYADLKDKDLLKAAS    465
TR6       ANNLVMRHVGDRMIISPPLTLTRDEIDLLIERAWKSLDEGMAEVKKQGLWQEG-    464
TR2       KNGLVMRAVGGTMIISPPLVLSREQVDELIDKARRTLDETHKAIGGA-------    457
TRCV      RNNLIMRACGDHIVSAPPLVMTRAEVDEMLAVAERCLEEFEQTLKARGLA----    459
TRAD      KNNLIMRACGDHIVSAPPLVISKEEIDQMLETAAKCMVEFEKQLKERGLV----    459
```

Fig. S3 Multiple sequence alignment of transaminases performed with Clustal Omega. Amino acids of the S-pocket are highlighted in green, amino acids of the L-pocket in blue and lysine, which binds PLP, is marked in yellow

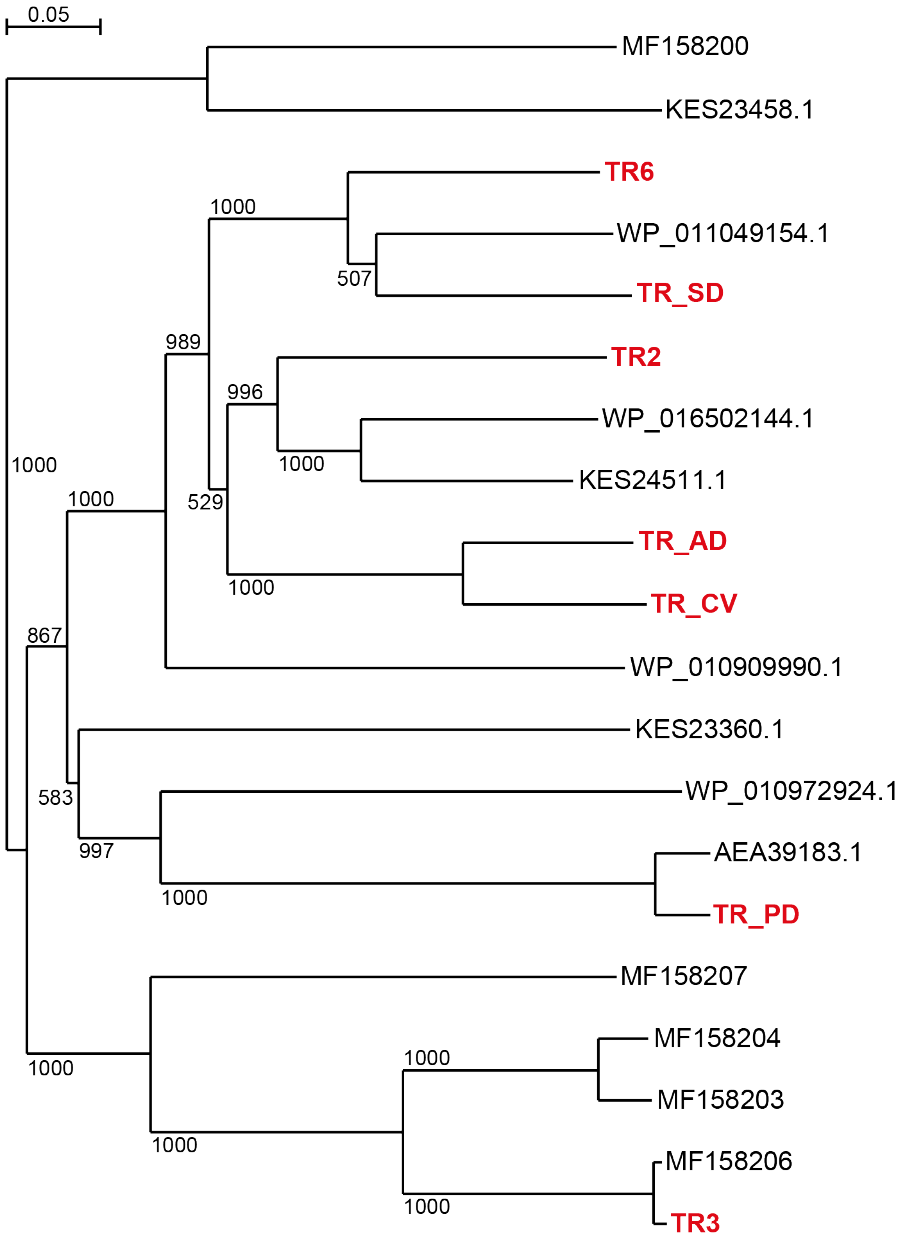

Fig. S4 Phylogenetic neighbor-joining tree of ω-transaminases. The bootstrap value was set to 1000. The following ω-TA's were taken for the tree: TR_AD: *A. denitrificans* (WP_159877958.1); TR_CV: *C. violaceum* (WP_011135573.1); TR_PD: *P. denitrificans* (ABL72050.1); TR_SD: *S. delicatus* (WP_093738538.1); TR1: *Pseudomonas* sp. (MF158200); TR2: *Acidihalobacter* sp. (MH588437); TR3: uncultured *Rhodobacteraceae* bacterium (MF158202); TR4: uncultured *Rhodobacteraceae* bacterium (MF158203); TR5: uncultured *Rhodobacteraceae* bacterium (MF158204); TR6: uncultured *Rhodobacteraceae* bacterium (MF158205); TR7: uncultured *Rhodobacteraceae* bacterium (MF158206); TR8: *Amphritea* sp. (MF158207); TR from *Pseudomonas* sp. AAC (KES23458.1, KES23360.1 and KES24511.1); TR from *Mesorhizobium loti* (WP_010909990.1); TR from *Vibrio fluvialis* (AEA39183.1); TR from *Pseudomonas putida* (WP_016502144.1); TR from *Agrobacterium tumefaciens* (WP_010972924.1) and TR from *Silicibacter pomeroyi* (WP_011049154.1)
